# Deciphering the tumor immune microenvironment of imatinib-resistance in advanced gastrointestinal stromal tumors at single-cell resolution

**DOI:** 10.1038/s41419-024-06571-3

**Published:** 2024-03-05

**Authors:** Xuechao Liu, Jing Yu, Yi Li, Hailei Shi, Xuelong Jiao, Xiaodong Liu, Dong Guo, Zequn Li, Yulong Tian, Fan Dai, Zhaojian Niu, Yanbing Zhou

**Affiliations:** 1 Department of General Surgery, Affiliated Hospital of Qingdao University, 16# Jiangsu Road, Qingdao, Shandong China; 2 School of Life Sciences and Biotechnology, Shanghai Jiao Tong University, Shanghai, 200240 China; 3 Pathology Department, Affiliated Hospital of Qingdao University, 16# Jiangsu Road, Qingdao, Shandong China; 4 Zhejiang Provincial Key Laboratory of Crop Genetic Resources, Institute of Crop Science, Plant Precision Breeding Academy, College of Agriculture and Biotechnology, Zhejiang University, Hangzhou, 310058 Zhejiang China

**Keywords:** Cancer microenvironment, Mechanisms of disease

## Abstract

The heterogeneous nature of tumors presents a considerable obstacle in addressing imatinib resistance in advanced cases of gastrointestinal stromal tumors (GIST). To address this issue, we conducted single-cell RNA-sequencing in primary tumors as well as peritoneal and liver metastases from patients diagnosed with locally advanced or advanced GIST. Single-cell transcriptomic signatures of tumor microenvironment (TME) were analyzed. Immunohistochemistry and multiplex immunofluorescence staining were used to further validate it. This analysis revealed unique tumor evolutionary patterns, transcriptome features, dynamic cell-state changes, and different metabolic reprogramming. The findings indicate that in imatinib-resistant TME, tumor cells with activated immune and cytokine-mediated immune responses interacted with a higher proportion of Treg cells via the TIGIT-NECTIN2 axis. Future immunotherapeutic strategies targeting Treg may provide new directions for the treatment of imatinib-resistant patients. In addition, IDO1+ dendritic cells (DC) were highly enriched in imatinib-resistant TME, interacting with various myeloid cells via the BTLA-TNFRSF14 axis, while the interaction was not significant in imatinib-sensitive TME. Our study highlights the transcriptional heterogeneity and distinct immunosuppressive microenvironment of advanced GIST, which provides novel therapeutic strategies and innovative immunotherapeutic agents for imatinib resistance.

## Introduction

Gastrointestinal stromal tumors (GISTs) are the most common mesenchymal tumors arising in the digestive tract. The majority of GISTs contain oncogenic mutations in either *KIT* (60–70%) or *platelet-derived growth factor receptor-α (PDGFRA)* (10–15%). About 15% of patients do not have *KIT* or *PDGFRA* mutations, but other genetic changes were observed in them. *PDGFRA* mutations mainly occur in exon 18 (90%), with fewer mutations in exons 12 and 14. The D842V mutation in exon 18 of *PDGFRA* accounts for about 50% of *PDGFRA* mutation [1–3]. With the advent of targeted therapy, the use of imatinib to target these mutant proteins has resulted in remarkable outcomes in advanced GIST. In recent decades, GISTs have become a paradigmatic and successful model in the emerging field of molecular-targeted therapies [4, 5].

The treatment of GIST should involve targeted genomic analysis or whole exome sequencing. When there is no mutation information, the conventional first-line imatinib, second-line sunitinib, and third-line regorafenib are recommended. The INTRIGUE study showed that ripretinib compared to sunitinib has a similar median PFS and better safety. Therefore, ripretinib can now be considered a second-line treatment after the standard dose of imatinib fails in advanced GIST [6]. Although imatinib can significantly improve prognosis, it rarely cures due to the emergence of tumor cells resistant to the drug. Imatinib resistance is divided into primary and secondary resistance. Primary resistance refers to disease progression within the first 6 months of treatment, accounting for about 10–15% of patients. Immediate resistance can be observed in about 10% of KIT/PDGFRA wild-type (WT) GISTs and 7-8% of tumors inherently resistant to imatinib due to primary mutations in KIT or PDGFRA (such as PDGFRA d842v mutation tumors). For patients with the PDGFRA d842v mutation, researchers have developed a new type of tyrosine kinase inhibitor, avapritinib. The results of two clinical studies, NAVIGATOR and VOYAGER, have well demonstrated its safety and effectiveness [7]. Secondary resistance denotes disease progression that reoccurs after more than 6 months of uninterrupted imatinib treatment, typically manifesting after more than 2 years of therapy. In clinical practice, approximately 40–50% of patients fall into this category. Studies have shown that imatinib-resistant GIST cases are frequently associated with secondary mutations, which are identified in over 80% of cases. The emergence of tumor clones with secondary mutations hinders imatinib binding, ultimately leading to tumor relapse [8–10]. With the occurrence of secondary mutations, almost all advanced patients eventually develop resistance to imatinib. With longer follow-up periods, imatinib resistance has emerged as a concerning clinical problem. As a result, overcoming drug resistance has become increasingly important for clinicians in the current era of adjuvant tyrosine kinase inhibitor (TKI) therapy [7–9].

For drug-resistant/metastatic refractory GISTs, small molecule inhibitors such as sorafenib and dasatinib have been tried in the treatment of GIST and have shown good therapeutic effects. The combination therapy targeting different points, including two types of tyrosine kinase inhibitors and the combination of tyrosine kinase inhibitors with downstream pathway inhibitors, is currently an important direction of exploration, offering hope for breaking through the treatment bottleneck of advanced GIST in the future [11]. Moreover, GISTs are a heterogeneous group of tumors with various molecular subtypes. As verified by many studies, this heterogeneity can result in significant variability of resistance among different tumor lesions, as well as within different regions of a single lesion. It has been established that the primary mechanism of resistance is the polyclonal expansion of cross-resistant subpopulations [12]. Despite being poorly explored in the management of GIST, preclinical data suggest that immunotherapy may be of interest, particularly in advanced GIST cases [13, 14]. Research shows that PD-1/PD-L1 inhibitors exert anti-tumor effects by rescuing exhausted CD8+ T cells in GIST through the blockade of the PI3K/AKT/mTOR signaling pathway [15]. In a clinical trial of stage III/IV GISTs patients treated with a combination of peginterferon α−2b (*PegIFNa2b*) and imatinib, a median follow-up of 3.6 years revealed that all 8 enrolled patients experienced a 100% remission in their condition. Furthermore, *PegIFNa2b* was found capable of inducing remission again in cases resistant to imatinib [16, 17]. Immunotherapy, as a new direction in cancer treatment, holds promise as a new hope for treating refractory and drug-resistant GISTs. Thus, there is a need for further research to investigate the potential role of immunotherapy in the management of GIST.

There has been growing evidence that immune evasion plays a crucial role in drug resistance and tumor progression, opening up the field of immunotherapy for GIST. The effectiveness of checkpoint immunotherapy depends largely on the tumor microenvironment (TME), which is characterized by a rich infiltrate of various immune cells and plays a pivotal role in controlling GIST [18]. Furthermore, diverse tumor-infiltrating immune cells are key players in tumor immune surveillance and immune evasion [19]. Thus, a comprehensive understanding of the TME is essential to comprehend the mechanisms behind tumor progression and drug resistance and, importantly, to facilitate the selection of innovative immunotherapeutic agents for advanced GIST. For traditional transcriptomic studies based on mixed cell populations, the resolution required to identify specific cell types is lacking and it it difficult to determine intratumoral heterogeneity and the TME. Recent advances in single-cell RNA sequencing (scRNA-seq) offer an unbiased analysis of molecular and cellular heterogeneity [20]. This approach has been widely employed to delineate the tumor multicellular ecosystem, including cancer-immune heterogeneity.

## Results

### The TME of GIST

**Fig. 1:**
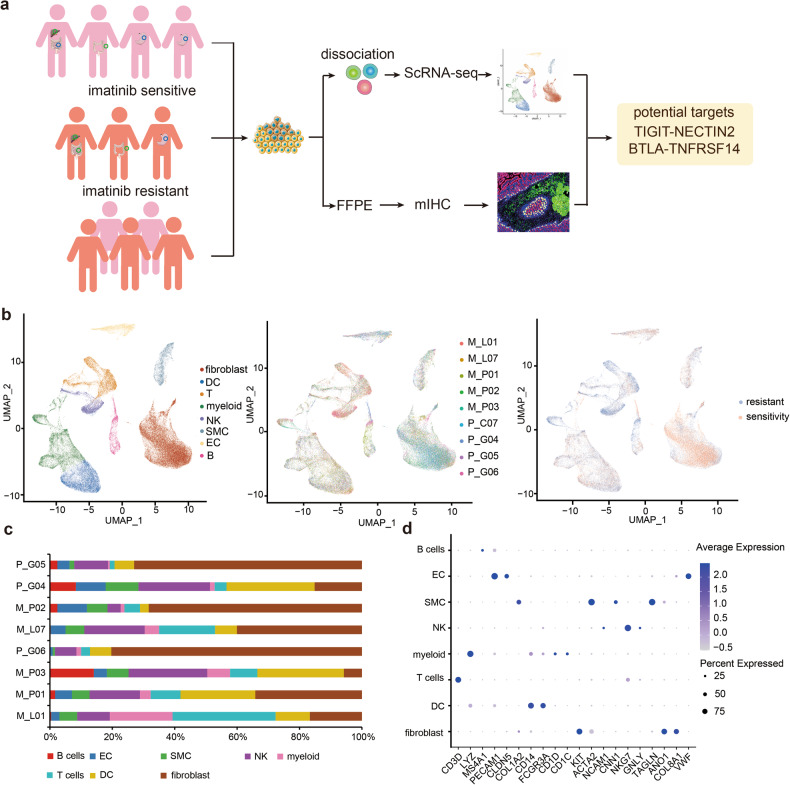
Single-cell transcriptome analysis of human GIST TME. **a** Overview of the study design. **b** UMAP plot of all cells from nine samples (five imatinib sensitive and four imatinib resistant) showing the composition of different cell types in human GIST TME. **c** Fraction of cells (y-axis) from each patient sample (x-axis) color-coded for cell type. **d** Dot plot showing marker gene expression for different cell types. EC endothelial cell, SMC smooth muscle cell, DC idendritic cell, NK natural killer, UMAP uniform manifold approximation and projection, mIHC multicolor immunofluorescence.

In order to comprehensively understand the tumor ecosystem in GIST, we obtained surgical tumor specimens from 7 GIST patients, with 4 specimens from 3 patients exhibiting imatinib resistance and 5 specimens from 4 patients exhibiting imatinib sensitivity. The scRNA-seq samples included primary, liver metastatic, and peritoneal metastatic GIST lesions. Six of the patients had previously received first-line targeted therapy with imatinib, and two had developed imatinib resistance, which progressed after third-line treatment. Furthermore, one locally advanced patient with PDGFRA exon 18 D842V mutation before first-line treatment was also included in the study. Detailed clinical and pathological information, including tumor stage, tumor size, treatment, and gene mutations were provided in Supplementary Data 1. Additionally, to validate the results, we recruited an additional 20 patients and performed immunohistochemical (IHC) and multiplex immunofluorescence (mIHC) staining (Fig. 1a). The IHC-sensitive group and the resistant group each consisted of 10 people, with mIHC staining performed on 5 people per group.

A total of 65,576 cells that passed the quality control stage, which were then visualized into 28 clusters using the uniform manifold approximation and projection (UMAP) method (Supplementary Fig. 1). These cells were classified into various subtypes, including fibroblast, DC (dendritic cells), T cells, myeloid, NK, SMC (smooth muscle cells), EC (endothelial cells) and B cells (Fig. 1b). It is noteworthy that all of these cell subtypes were shared among patients (Fig. 1b) and between imatinib-resistant and sensitive, albeit at varying proportions (Fig. 1b, c). Moreover, marker gene expression analysis for each cluster revealed that B cells highly expressed MS4A1; EC highly expressed PECAM1 and CLDN5; SMC highly expressed COL1A2, ACTA2, CNN1, and TAGLN; NK cells highly expressed NKG7 but not CD3D; myeloid highly expressed LYZ; T cells highly expressed CD3D, DC highly expressed CD14 and FCGR3A; fibroblast highly expressed KIT, ANO1, and COL8A1, which were marker genes for GIST (Fig. 1d).

### Analysis of the tumor evolution of GIST

Since SMC-expressed marker genes of fibroblasts, including COL1A2, and ACTA2 were expressed in both these cells and fibroblasts (Fig. 1d), this work extracted both types of cells from the single-cell sequencing data for inferCNV analysis. Observation showed that the copy number changed dramatically in fibroblasts but slightly in smooth muscle cells (Supplementary Fig. 2). To further confirm the tumor cells in tissues, we calculated the chromosome ploidy of fibroblasts and smooth muscle cells using the copyKAT algorithm, finding that the latter had normal ploidy (Supplementary Fig. 3), based on which, we selected fibroblasts for subsequent analysis.

**Fig. 2:**
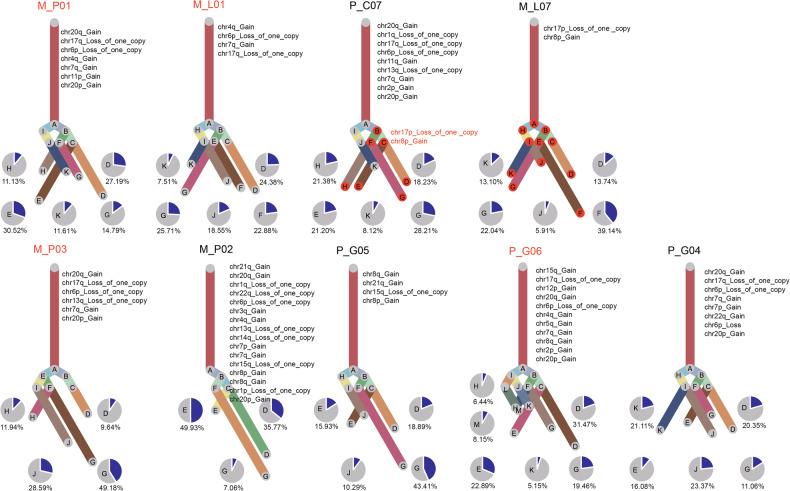
Clonal evolution analysis of GIST cells. The branches are delineated according to the percentage of cells in the subclone containing the corresponding CNVs. The canonical CNV events in each lesion were labeled in the clonality tree. The sample name colored by red means imatinib resistant samples, while the ones colored by black means imatinib sensitive samples. The subclones in red background indicated the shared subclone between two samples from the same patient.

First, we analyzed the tumor evolution in different samples and listed each sample’s clonal copy number variation at the trunk of the phylogenetic tree. Thereinto, the most common mutations included 7q gain, 20p gain, and 17q loss of one copy, which was consistent with the previous results of GIST genome sequencing [21]. Furthermore, the M_L07 and P_C07 samples were metastatic and primary lesions of the same patient, respectively, and their common CNV region was analyzed herein. Results show that all the tumor cells in the M_L07 sample present the mutations of 17p loss of copy and 8p gain, which evolved from the B subclone in the P_C07 sample. This indicates that the metastatic lesion is formed by seeding the primary one in earlier stages, and the tumor cells of both lesions experience the tumor evolution separately (Fig. 2; Supplementary Data 2). Further, in both of them, the GIST is featured with high intratumoral heterogeneity in DNA level.

### Transcriptional heterogeneity of tumor cells

**Fig. 3:**
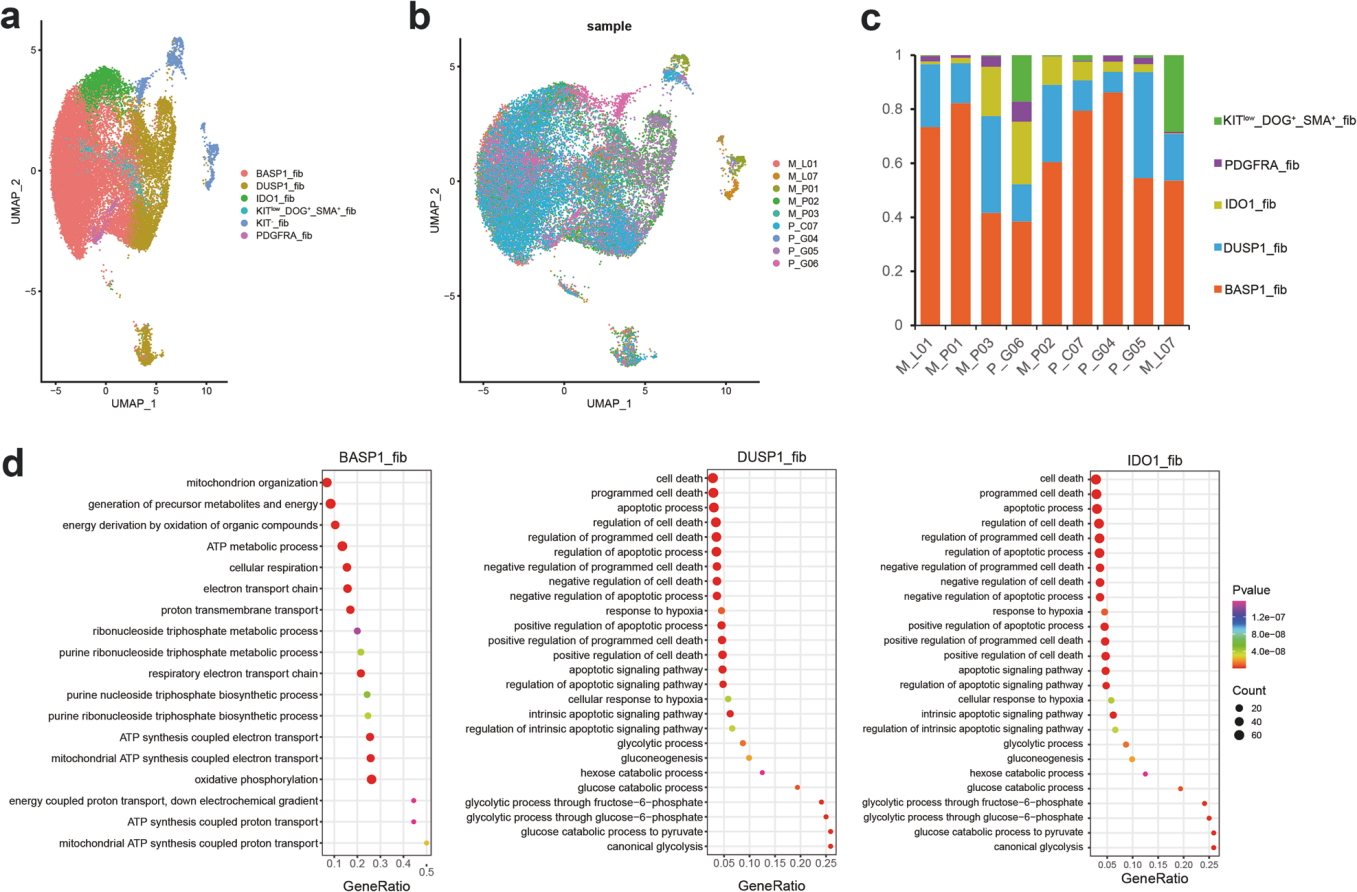
Transcription characteristics and heterogeneity among the six malignant cell types. **a** UMAP analysis showing the six cell type of malignant cells. **b** Colored cells by each sample. **c** Bar plot showing the cell count proportion of each maligant subcluster in GIST. **d** GO enrichment analysis of marker genes form BASP1_fib (left), DUSP1_fib (middle), IDO1_fib (right).

We performed subdivided cluster analysis on the selected fibroblasts, resulting in the identification of 15 distinct subpopulations (Supplementary Fig. 4a). Based on the gene expression profile of each subpopulation, we classified the tumor cells into several categories, including brain acid-soluble protein 1 (BASP1)_fib, dual specific phosphatase 1 (DUSP1)_fib, indoleamine 2,3-dioxygenase 1 (IDO1)_fib, KIT low DOG^+^ SMA^+^_fib, PDGFRA_fib, and KIT^-^_fib, which were observed in all samples (Fig. 3a, b; Supplementary Fig. 4b). Thereinto, KIT^-^_fib was more distant from other cells in the UMAP plot, indicating its significantly different function from others. We analyzed the expression level of KIT in fibroblasts for KIT overexpression is a marker of GIST [22], with a finding that KIT was highly expressed in fibroblasts except for subclusters10 and 11 (Supplementary Fig. 4b). Considering the lower proportion of KIT^-^ in GIST, we also studied the chromosome ploidy of each subpopulation and found that the ploidy was normal in KIT^-^ cells, but abnormal in KIT^+^ cells (Supplementary Fig. 4e). On this basis, we proposed that the KIT^-^ cells may be normal fibroblasts, while the KIT^+^ ones were all tumor cells.

PDGFRA, a key driver gene in GIST and known to have mutations associated with imatinib resistance [23, 24], was highly expressed in a specific cell subpopulation named PDGFRA_fib (Supplementary Fig. 4b). Similarly, the gene BASP1 was highly expressed in the BASP1_fib subpopulation (Supplementary Fig. 4b). It promoted the growth of tumor cells and acted as an adverse prognosis factor in lung adenocarcinoma, pancreatic cancer, and tongue squamous cell carcinoma [25–27]. DUSP1_fib highly expressed DUSP1 (Supplementary Fig. 4b), which was a gene highly expressed in tumor cells, showing drug resistance in multiple tumors, including gefitinib resistance in non-small cell lung cancer, apatinib resistance in gastric cancer, paclitaxel resistance in ovarian cancer, gemcitabine resistance in gallbladder carcinoma, etc [28–31].

Firstly, we analyzed the expression of PD-L1 (CD274) in each tumor subpopulation. PD-L1 expression was extremely low in all samples, no matter whether imatinib-resistant ones or sensitive ones (Supplementary Fig. 4d). This result suggested that anti-PD-1 or anti-PD-L1 therapy might be not the best choice in the second-line treatment for imatinib-resistant GIST patients. The high expression of IDO1, a gene expressed in KIT^+^ GIST tumor cells, was identified as an important mechanism for imatinib resistance [32]. Imatinib achieves its tumor-killing effect by inhibiting IDO1 expression through the KIT pathway. In this work, a certain number of highly expressed IDO1 tumor cells were identified and named IDO1_fib (Supplementary Fig. 4b). In addition, since some cells with a slightly lower expression level of KIT highly expressed DOG1, which is another driver gene of GIST [23], we named them as KIT^low^ DOG^+^ SMA^+^_fib (Supplementary Fig. 4b). To sum up, BASP1_fib and DUSP1_fib were the most common cell subpopulations in GIST (Fig. 3c).

Then, we analyzed the functions of all the above-mentioned cell subpopulations. The normal KIT^-^ fibroblasts enriched a series of immune-associated GO, including monocyte chemotaxis, myeloid leukocyte migration, myeloid leukocyte activation, and leukocyte-mediated immunity. BASP1_fib highly enriched the oxidative phosphorylation and its pathway (Fig. 3d), while DUSP1_fib and IDO1_fib highly enriched the glycolytic pathway (Fig. 3d), which indicated that the tumor cells in GIST underwent different metabolic reprogramming. Besides, the pathways, including cell death-related ones, were highly enriched in DUSP1_fib and IDO1_fib (Fig. 3d) but not in BASP1_fib, implying the significantly different functions of tumor cells passing through varying metabolic pathways. These results suggested the GIST is featured with high intratumoral heterogeneity intranscription level.

Next, we studied the difference of all tumor cell subpopulations between imatinib-resistant and imatinib-sensitive groups and performed the reactome enrichment analysis on differently expressed genes. The reactome pathways with similar functions were included in the same module, including immune, interferon, cytokine, etc., with each module’s signal pathways listed in Supplementary Data 3. In the imatinib-resistant group, the interferon-associated reactome pathway was significantly enriched in each subpopulation’s up-regulated genes rather than down-regulated ones (Supplementary Fig. 5a), while the immune- and cytokine-associated reactome pathways were slightly enriched in down-regulated genes but significantly enriched in up-regulated ones (Supplementary Fig. 5b). On this basis, we believed it was the imatinib-resistant tumor cells in GIST that activated the immune- and cytokine-mediated immune responses.

The pathways included in the PDGFR module were all related to TKI resistance, including imatinib-resistant PDGFR mutants, sunitinib-resistant PDGFR mutants, regorafenib-resistant PDGFR mutants, sorafenib-resistant PDGFR mutants, and PDGFR mutants bind TKIs. Since the PDGFR pathway was enriched in each subpopulation’s highly expressed genes in the imatinib-resistant group, it was deduced that the imatinib resistance in GIST was associated with PDGFR mutants. In the same group, the hypoxia-associated reactome pathway was more significantly enriched in up-regulated genes of DUSP1_fib and IDO1_fib (Supplementary Fig. 5a) that took glycolysis as a metabolic pathway. Meanwhile, the hypoxia pathway was enriched in the up-regulated genes of BASP1_fib, although this subpopulation activated the oxidative phosphorylation pathway (Supplementary Fig. 5a), which implied that the imatinib resistance in GIST was correlated with hypoxia.

In this imatinib-resistant group, the Receptor Tyrosine Kinases, p53 pathway, PTEN pathway, WNT pathway, NOTCH pathway, and cell death pathway were more significantly enriched in up-regulated genes of BASP1_fib and DUSP1_fib (Supplementary Fig. 5a), but in down-regulated genes of PDGFRA_fib, IDO1_fib, and KIT^low^ DOG^+^ SMA^+^_fib (Supplementary Fig. 5b), which indicated that the drug-resistance mechanism varied with different cell subpopulations in GIST.

### Analysis of the immune microenvironment of GIST

**Fig. 4:**
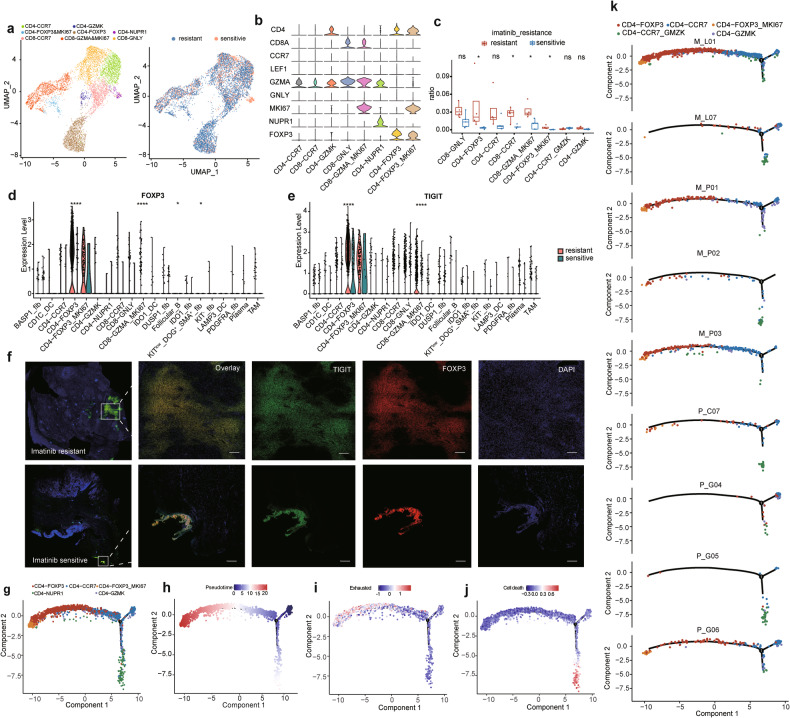
The heterogeneity within the T cells. **a** 8 subclusters of CD4 T cells were identified by UMAP analysis. **b** Marker gene expression of each cell type. **c** Comparison of cell count proportion of each cell type between imatinib resistant and sensitive patients. **d**, **e** Expression of FOXP3 and TIGIT in each cell type split by imatinib resistant and sensitive patients. **f** The coexpression of FOXP3 (red) and TIGIT (green) by polychromatic immunofluorescence method in imatinib resistant and sensitive patients. The Monocle 2 trajectory plot showing the dynamics of CD4 T cell subclusters (**g**) and their pseudotime curve (**h**). The exhausted signature (**i**) and cell death signature (**j**) expression along trajectory pathway. **k** The trajectory plot showing dynamics of CD4 T cell subcluster splited by different samples.

The immune microenvironment of each sample was explored to further analyze the imatinib-resistance mechanism in GIST. First, we categorized the T cells into the following subpopulations: CD4-CCR7, CD4-GZMK, CD4-FOXP3&MKI67, CD4-FOXP3, CD4-NUPR1, CD8-CCR7, CD8-GZMA&MIK67, and CD8-GNLY (Fig. 4a, b). We compared the cell proportion between imatinib-resistant and imatinib-sensitive patients and between primary and metastatic lesions (Fig. 4c and Supplementary Fig. 6a). Surprisingly, imatinib -resistant patients had a much higher proportion of Treg cells (CD4-FOXP3) than imatinib -sensitive ones, with the lowest level in the former higher than the highest level in the latter, which implied the possible participation of Treg cells in imatinib resistance. Besides, the number of proliferating Treg cells (CD4-FOXP3&MKI67) also dramatically increased in imatinib-resistant patients, while that of cytotoxic CD8 T cells plummeted (Fig. 4c). However, there was no obvious difference after standardizing the total cellular score even if the metastatic lesion contained more Treg cells (Supplementary Fig. 6a). The above results further verified the correlation between Treg cells and imatinib resistance.

The gene expression analysis on these cells showed that the TIGIT, an immune checkpoint gene participating in the tumor immune escape, was highly and specifically expressed in Treg cells and proliferating Treg cells, and TIGIT showed differential expression in Treg cells between drug-resistant patients and drug-sensitive ones (Fig. 4d, e) [33, 34].

To further prove the effect of Treg cells on imatinib resistance, we performed immunohistochemical (IHC) analysis on a queue containing 10 imatinib-resistant and 10 imatinib-sensitive samples. The expression of FOXP3, which was a marker gene in Treg cells, increased markedly in imatinib-resistant samples (*P* < 0.05; Supplementary Fig. 6b). We also measured the coexpression of FOXP3 and TIGIT by polychromatic immunofluorescence method and observed that both genes were expressed in the same cell (Fig. 4f; Supplementary Fig. 7a). These results implied the high enrichment of TIGIT+ Treg cells in the former, based on which we conjectured that the application of target TIGIT or target Treg may provide new therapeutic directions for imatinib resistance.

Next, the dynamic immune states and cell transitions in CD4+ T cells were explored using Monocle2 to perform trajectory inference, and the results showed that the CD4-CCR7 cells were at the beginning of the trajectory path, termed as state 3 (Fig. 4g). CCR7 is a marker gene of naïve T cell; and accordingly, subcluster CD4-CCR7 showed enrichment of GO term related to T cell differentiation, such as T-helper cell differentiation, alpha-beta T cell differentiation (Supplementary Fig. 6c). In addition, CD4-CCR7 cells were separated into two states, suggesting the differential function between these cells. The two terminal portions of the trajectory were CD4-FOXP3 cells, followed by CD4-FOXP3&MKI67 cells, termed state2, and CD4-NUPR1, termed state 1 (Fig. 4g, h). Cells that are CD4-FOXP3&MKI67 positive showed significant enrichment in GO terms related to cell cycle processes, including the mitotic cell cycle and cell cycle phase transition. This implies a high level of proliferative activity in these cells. (Supplementary Fig. 6c). CD4-FOXP3 and CD4-GZMK highly enriched the immune-related GO terms, consistent with the canonical immune cell type’s function (Supplementary Fig. 6c). We analyzed the exhausted signature (Supplementary Data 4) along the trajectory, showing the highest level in the terminal of state2 (Fig. 4i). The expression of FOXP3 and TIGIT were highest at the terminal of the trajectory path, consistent with the exhausted signature (Supplementary Fig. 6e, f). CD4-NUPR1 cells were enriched in cell death relate GO, such as apoptotic signaling pathway, programmed cell death (Supplementary Fig. 6c), and genes involved in cell death, such as NUPR1, PDE1A and TIMP3 showed the highest expression at the terminal of state 1 Supplementary Fig. 6g-i). We analyzed the cell death signature (Supplementary Data 4) along the trajectory, cell death was enriched in the terminal of state 1 (Fig. 4j).

Then, the different cell densities for each patient along the trajectory path are analyzed. The resistant samples contained more cells along state 2 (Fig. 4k), consistent with the high enrichment of treg cells and proliferated Treg cells (Fig. 4c). The proportion of CD4-CCR7 cells in state 2 significantly increased in imatinib-resistant patients, while that in state 3 dramatically increased in imatinib-sensitive patients and the No. 6 patient with primary resistance to this drug (Supplementary Fig. 6j). The CD4-CCF7 cells presented cytotoxic characteristics in their enriched region under state 2 (Supplementary Fig. 6k), where the killer cells, GZMA and GZMK, were highly and significantly expressed (Supplementary Fig. 6l, m). This indicates that the naïve T cells, with certain characteristics of killer T cells in imatinib-resistant patients, are more likely to evolve into suppressor Treg cells. In a word, under the promotion of imatinib resistance, the CD4+ T cells can evolve into suppressor T cells, and may eventually experience apoptosis among imatinib-sensitive patients.

In analyzing B cells in the tumor microenvironment, B cells were separated into two clusters: one termed as follicular B cell, with high expressions of MS4A1, CD79B, CD52, and another termed as plasma cell, with high expressions of IGHG1, IGHG4 and MZB1 (Supplementary Fig. 8). While the cell numbers of distinct clusters varied significantly among patients (Fig. 1d), our analysis led us to conclude that B cells might be not the critical cell type for imatinib resistance.

**Fig. 5:**
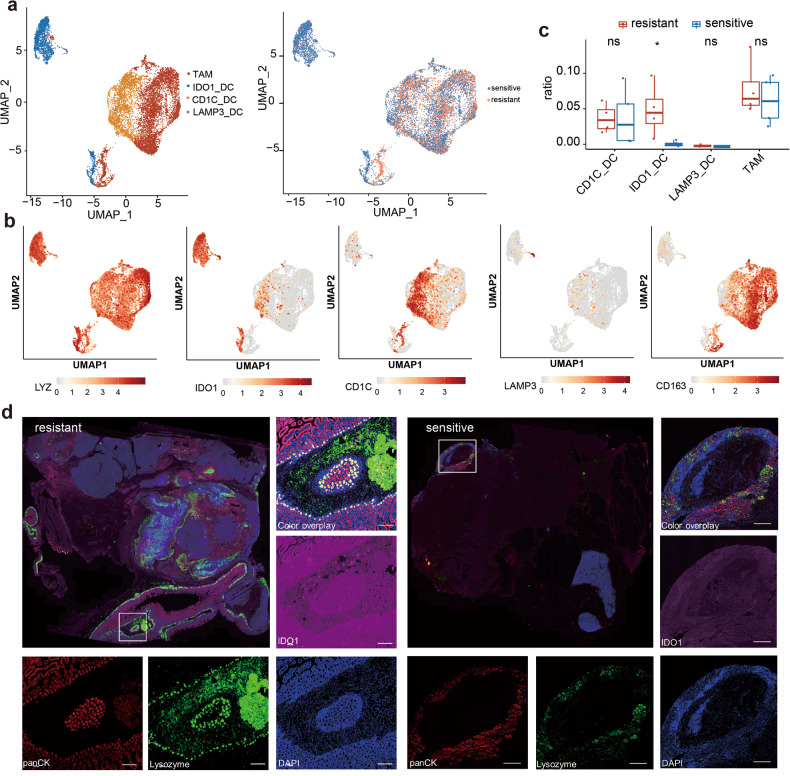
The heterogeneity within the myeloid cells. **a** 4 subclusters of myeloid cells were identified by UMAP analysis. **b** Marker gene expression of each cell type. **c** Comparison of cell count proportion of each cell type between imatinib resistant and sensitive patients. **d** mIHC staining of panCK (red), LYZ (green), IDO1 (magenta) and DAPI in GIST TME.

Then, we analyzed the myeloid cells and classified them into several subpopulations, including TAM, IDO1_DC, CD1C_DC, and LAMP3_DC, which all highly expressed their marker gene LYZ (Fig. 5a, b). Thereinto, IDO1_DC specifically expressed IDO1; CD1C_DC, which is probably a kind of conventional DCs, specifically expressed CD1C [35]; LAMP3_DC, which may be a mature form of conventional DCs, highly expressed LAMP3 (Fig. 5b). It is worth noting that the IDO1_DC, with an increased proportion in imatinib-resistant patients, may have a relationship with imatinib resistance (Fig. 5c). We performed multicolor immunofluorescence (mIHC) to identify the IDO1_DC distribution in TME. IDO1_DC was largely more distributed in the imatinib-resistant TME than in sensitive TME (Fig. 5d; Supplementary Fig. 7b).

### Intercellular communication in the tumor microenvironment of GIST

**Fig. 6:**
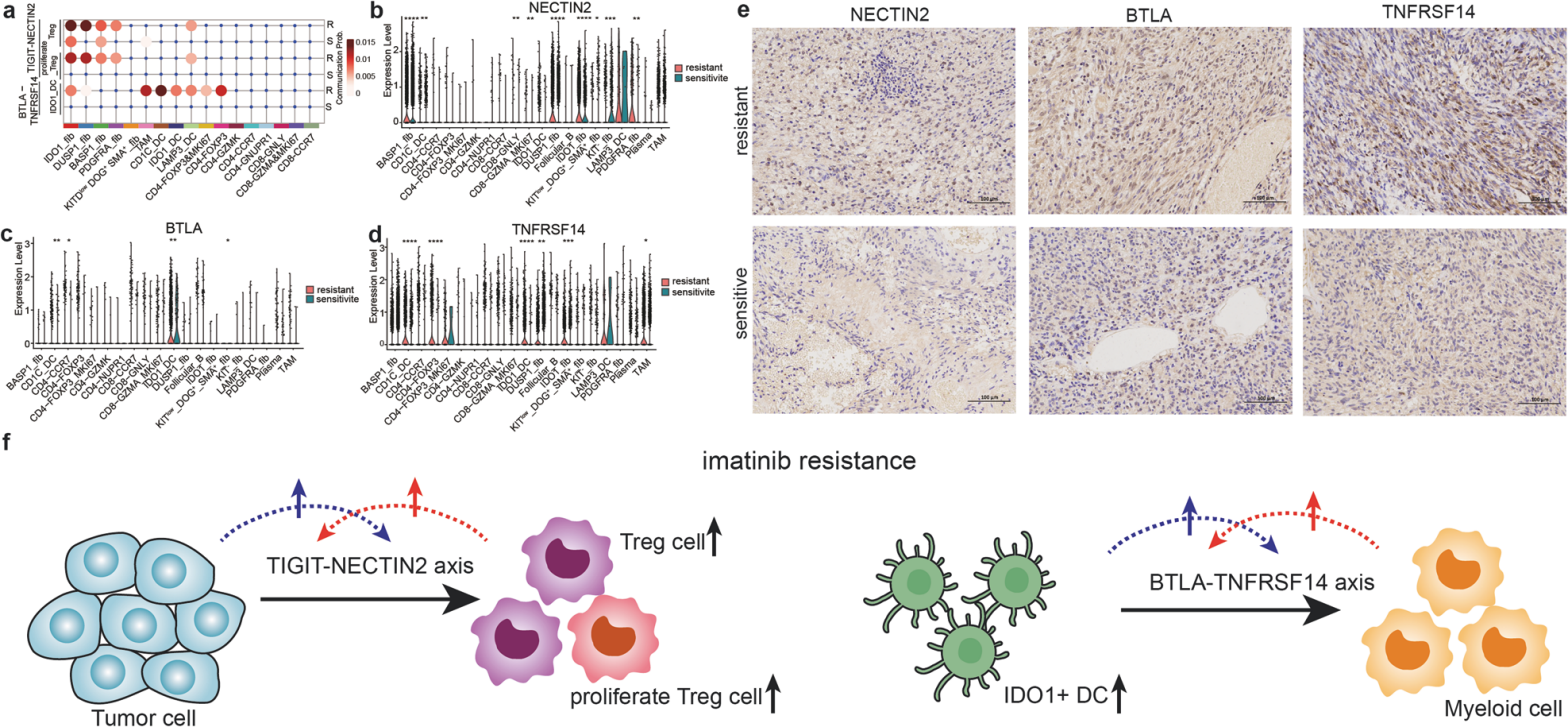
The comprehensive immunosuppressive mechanism in imatinib resistant GIST. **a** Cell communication analysis on TIGIT-NECTIN2 and BTLA-TNFRSF14 pair between different cell types in imatinib resistant and sensitive patients respectively. Expression of NECTIN2 (**b**), BTLA (**c**) and TNFRSF14 (**d**) in each cell type in imatinib resistant and sensitive patients respectively. **e** IHC analysis of NECTIN2, BTLA and TNFRSF14 between imatinib resistant (upper) and sensitive (bottom) patients. **f** Schematic diagram of the unique tumor-immune microenvironment of imatinib-resistance in advanced GIST.

We probed into the molecular mechanism of imatinib resistance in GIST by analyzing the intercellular communication. In general, tumor cells underwent the outgoing interaction most frequently, while Treg and TAM cells contained the largest number of received signals (Supplementary Fig. 9). Then, we researched the difference in intercellular communication in different tumor cell subpopulations between imatinib-resistant and imatinib-sensitive patients. Results showed that there were four subpopulations, namely, PDGFRA_fib, BASP1_fib, DUSP1_fib, and IDO1_fib, experiencing intercellular communication with Treg and proliferating Treg cells through TIGIT-NECTIN2 in imatinib-resistant patients, while in imatinib-sensitive ones, only BASP1_fib and IDO1_fib weakly communicated with Treg cells. In addition, intercellular communication also occurred between IDO1_DCs and myeloid cells through BTLA-TNFRSF14 in imatinib-resistant patients, which was not significant in imatinib-sensitive ones (Fig. 6a).

Among patients with imatinib resistance, TIGIT was highly expressed in Treg and proliferating Treg cells (Fig. 4e), NECTIN2 was highly expressed in tumor cells and LAMP3_DC (Fig. 6b); BTLA was highly expressed in IDO1_DCs (Fig. 6c); TNFRSF14 was highly expressed in myeloid cells and tumor cells, especially in the imatinib-resistant patients (Fig. 6d). Although violin plots show high expression of TNFRSF14 in CD4-FOXP3&MKI47 and LAMP3_DC cells in sensitive patients, only three cells from these two categories express TNFRSF14 in sensitive patients. Therefore, the CellChat analysis does not calculate communication between the two. The gene expression of the above pairs indicated that the activation of immune checkpoint pathways participated in the imatinib resistance. The IHC analysis on clinical samples also proved the increased expression of immune checkpoint proteins in these patients (Fig. 6e).

## Discussion

GIST is a highly heterogeneous tumor including various molecular entities with mutually exclusive gain-of-function mutations in *KIT* or *PDGFRA* mostly. The therapeutic approach for advanced GIST has been transformed by imatinib, resulting in substantial reductions in recurrence and metastasis risks, and a remarkable improvement in clinical outcomes for patients. The median overall survival rate has increased from 18 months to over 70 months due to the drug’s significant impact. However, resistance to imatinib is usually unavoidable in clinical practice: approximately 10–15% of GIST patients have primary resistance to it [36, 37]; about 40–50% of patients have secondary resistance to it, which often occurs more than two years after treatment [8, 38]. Since imatinib resistance can result in poor prognosis, it has become a pressing problem faced by clinicians and also a research focus among researchers.

In recent years, immunotherapy has presented its clinical benefit in the treatment of multiple solid tumors, but its effect on GIST remains unclear. Consequently, investigating the immune microenvironment of tumors and identifying effective therapeutic targets has become a crucial research direction to overcome drug resistance and enhance immune efficacy. Based on the single-cell sequencing, we analyzed the cellular blueprint and immune microenvironment of advanced GIST, including primary, peritoneal metastatic, and liver metastatic lesions, and summarized the transcriptional heterogeneity of imatinib resistance in this disease, which may provide important directions for the future treatment of GIST.

Based on the analysis on single-cell data, the tumor cells can be divided into six categories, i.e., PDGFRA_fib, BASP1_fib, DUSP1_fib, IDO1_fib, KIT^low^ DOG^+^ SMA^+^_fib, and KIT^-^_fib, of which BASP1_fib and DUSP1_fib are the most common cell subpopulations in GIST (Fig. 3). BASP1 belongs to the family of growth proteins and studies have found that it has multiple mechanisms of action in tumors, such as abnormal modification of promoter methylation, promoting the signaling of EGFR, and axonal growth and development. BASP1 is involved in the activation of the Wnt pathway in various tumors, thereby regulating the cell cycle and promoting cell proliferation. It has been reported that BASP1 is an adverse prognostic factor, because its overexpression in lung adenocarcinoma tissues promotes the proliferation and migration of cancer cells, which is closely related to poor prognosis [27]. Similarly, DUSP1 is also resistant to multiple antitumor drugs. DUSP1, as an important member of the Mitogen-activated protein kinase (MAPK) gene family, is considered a tumor suppressor and a regulator of cancer-related inflammation. The functions of DUSP1 are focused on cell proliferation, differentiation, stress response, cycle arrest, and apoptosis, mainly realized through the regulation of the MAPK signaling pathway. Studies have shown that DUSP1 is involved in the regulation of the occurrence and development of various tumors. Sanders et al., have verified that the expression of this gene is directly correlated with drug resistance and positively correlated with prognosis in high-grade serous ovarian carcinoma [39]. We speculated that the high proportion of tumor cells expressing BASP1 or DUSP1 might be one of the reasons for the poor prognosis of advanced GIST. Therapies targeting BASP1 or DUSP1 might be a good choice for future GIST treatment strategies.

Moreover, we identified a certain number of highly expressed IDO1 tumor cells and named them IDO1_fib (Fig. 3). It is important to note that our analysis of the subpopulations of myeloid cells and their specifically expressed genes revealed a significant increase in the number of IDO1_DCs in imatinib-resistant patients (Fig. 5). This observation suggests a close association between these cells and imatinib resistance. IDO1 is a rate-limiting enzyme in the extracellular tryptophan catabolism pathway via kynurenine, and it has been proven to be abnormally highly expressed in malignant tumors such as cervical cancer and esophageal cancer. It is involved in tumor immune evasion and promotes tumor growth and distant metastasis. Studies have reported a positive rate of about 89.8% for IDO1 in GIST [40]. It generates and activates the Treg and myeloid-derived suppressor cells by inhibiting T and NK cells, thus promoting tumor angiogenesis [41, 42]. However, as verified by Balachandran et al., imatinib can enhance the antitumor effect by activating the CD8+ T cells and inducing the T-reg apoptosis through IDO1 inactivation [32]. Therefore, the IDO1 inhibitors may provide a new direction for the immunotherapy of GIST in drug-resistant patients.

The analysis of tumor evolution shows that the metastatic lesion is formed by seeding the primary one in earlier stages, and the tumor cells of both lesions experience the tumor evolution separately (Fig. 2). Wang et al. reported that compared to linearly evolved tumors, the parallelly evolved ones usually shared the finite mutation between primary and metastatic clones and recurred more rapidly after the first operation, which was in line with our study [43]. Zhou et al. analyzed the single cells of osteosarcoma and found that there was more CNV in major clones and primary lesions, but less CNV in subclones and recurrent lesions, which was not consistent with our conclusions drawn by comparing the CNV between samples [44]. This is probably because the tumor cells are significantly different between GIST samples, and tumors are highly heterogeneous between primary and metastatic lesions. Under such circumstances, the TKI drugs, for example, imatinib, finally become resistant to drugs after the clonal selection of tumor cell subclones. Once the body becomes drug-resistant due to the mutation resulting from subclones, the tumor cells will proliferate rapidly to form new tumors during treatment. Meanwhile, the high tumor heterogeneity also makes the follow-up treatment more difficult.

According to the pathway enrichment analysis, tumor cells are greatly different in functions after experiencing various metabolic reprogramming. As the tumor grows, its metabolic characteristics will get differentiated, thus causing significant heterogeneity in tumor metabolism (Fig. 3). This work highlights the intratumoral heterogeneity of GIST and the signal pathways that may drive the progression and recurrence of this disease. By analyzing the difference between imatinib-resistant and imatinib-sensitive groups, we found that the tumor cells of GIST in the former group activated the immune- and cytokine-mediated immune responses and the interferon-associated reactome pathway was significantly enriched in up-regulated genes of each tumor cell subpopulation (Supplementary Fig. 5). Previous research has indicated that interferon can support the tumor immune surveillance and escape by guiding the metabolic reprogramming of tumor cells, which implies that each subpopulation can create an immunosuppressive microenvironment while driving tumors’ resistance to drugs [45, 46]. Besides, the drug-resistant subpopulations can enrich different pathways, which may lead to various drug-resistance mechanisms. It is worth noting that the hypoxia pathway’s enrichment in these subpopulations of GIST implies the possible correlation between imatinib resistance and hypoxia. Compared to sensitive tumors, drug-resistant tumors may exhibit a stronger preference for nutrient depletion and the selection of hypoxic survival strategies. This state of tumor heterogeneity serves as the foundation and safeguard for tumors to survive drug pressure and further develop into dominant drug-resistant clones. Xu et al. concluded that the metabolic phenotype of GIST could be changed by ROS and HIF-1α during the long-term use of imatinib, which may promote resistance to this drug [47]. They believed that new metabolic targets were potentially effective strategies to overcome the drug resistance in GIST. Furthermore, it has been proved in vitro experiments that the newly developed pimitespib, which is a heat shock protein 90 (HSP90) inhibitor, can cooperate with sunitinib to solve the same problem by inhibiting the protein kinase D2 and hypoxia-inducible factor-1 alpha.

Tumor heterogeneity refers to changes in molecular biology or genetics during tumor progression, resulting in differences in growth rate, invasive ability, and drug sensitivity among varied tumor cells. In our study, both tumor evolution and transcriptional analyses have shown tumor heterogeneity. Intratumoral heterogeneity refers to the existence of diversity among different tumor cells within the tumor, such as the coexistence of different subgroups of tumor cells with high expression of different genes, whose interactions can better maintain a balanced state of tumor heterogeneity [48]. Different tumor cell subgroups have undergone different metabolic reprogramming, resulting in different functions. Additionally, tumor evolution analysis found that metastatic tumors and primary tumors have genetic diversity, reflecting the spatial heterogeneity of the tumor.

The outcome of immunotherapy depends on the tumor microenvironment, which is primarily constituted by tumor-infiltrating immune cells that not only regulate the immune response of local tumors but also act as important targets of this therapy. To further analyze the imatinib-resistance mechanism in GIST, we explored the immune microenvironment of this disease by single-cell sequencing, based on which eight T-cell subpopulations were identified. As shown by the analysis of their difference, the proportion of Treg cells (CD4-FOXP3) in drug-resistant patients was much higher than that in drug-sensitive ones, and even the lowest level of the former was higher than the highest level of the latter. By contrast, the number of cytotoxic CD8+ T cells plummeted in drug-resistant patients. The M_G06 sample, which had primary drug resistance and was not treated with imatinib, still contained a higher proportion of Treg cells (Fig. 4).

The analyses on IHC, mIHC showed that the Treg infiltration increased significantly in drug-resistant patients, implying the possible participation of Treg cells in imatinib resistance. In combination with the trajectory analysis, we found that the naïve T cells, which presented certain characteristics of killer T cells in these patients, were more likely to evolve into suppressor Treg cells. The Treg cells highly and specifically expressed TIGIT, a gene participating in the tumor immune escape and playing a key role in the pathological process of tumor progression as an inhibitory receptor indispensable to immunoregulation (Fig. 4). The application of target TIGIT or target Treg may provide new directions for the treatment of imatinib-resistant patients. According to existing clinical studies with favorable results, TIGIT is expected to combine with PD-1/PD-L1 inhibitors to block its pathway, thus enhancing the body’s immune response to cancer cells and improving the antitumor activity [49].

Thereinto, the PD-L1 expression is up-regulated in multiple tumors, and the combination of PD-1 with its ligand PD-L1 can inhibit the activated immune cells to induce the tumor immune escape. Previous studies have shown that PD-L1 expression is higher in low-risk and non-recurrent metastatic samples [50]. By comparing the gene expression profiles before and after the treatment of GIST with imatinib, it is observed that imatinib can down-regulate the PD-L1 expression by inhibiting KIT and PDGFRA, which countervails the immunosuppression of GIST [18]. In this work, PD-L1 was lowly expressed in each sample, showing no obvious difference between groups (Supplementary Fig. 4d), which, based on our conjecture, may be a crucial reason for the poor outcome of traditional immune checkpoint inhibitors.

We constructed a cell-cell interactome landscape to further analyze the molecular mechanism of imatinib resistance in GIST. Treg and tumor-associated macrophage (TAM) are cells containing the largest number of received signals. Previous research shows that TAM, which is a type of important immune cell in GIST, can be differentiated into different subtypes in a specific microenvironment, and its number is closely related to tumor recurrence and prognosis [51, 52]. In this work, we concluded similar to that drawn by Mao et al., who performed the single-cell analysis on two patients who suffered from low- and high-risk GIST, respectively. They found that macrophages, located in the center of the tumor microenvironment and most affected by other cell signals, helped build a relationship between tumor cells and other cell types [53]. After repeated confirmation, we discovered that the number of TAMs had no obvious difference between drug-resistant and non-resistant samples and between metastatic and non-metastatic samples.

In addition, the intense intercellular interaction between tumor and immune cells forms a favorable environment for imatinib resistance. Gonzalez et al. found that the BTLA expression on the surface of NK cells was correlated with the poor prognosis of patients with chronic lymphocytic leukemia (CLL). They first revealed the inhibitory effect of BTLA/HVEM on NK cell-mediated immune responses and the influence on patients’ prognosis in this disease, suggesting that the BTLA/HVEM axis may be a potential therapeutic target for CLL [54]. As one of the most promising targets in recent years, TIGIT is primarily expressed on the surface of T and NK cells. It inhibits the immune cells in multiple steps of tumor immune circulation and has been proved to be a key factor in inhibiting the adaptive and innate immunity of tumors [55]. The TIGIT-NECTIN2 axis can regulate the immunosuppressive environment and intercellular interaction in liver cancer, providing mechanism information for the effective treatment of this cancer [56]. In our study, we performed single-cell sequencing on tumor tissues from patients exhibiting both imatinib-resistant and imatinib-sensitive GISTs. This allowed us to identify distinct cell types within the TME, and to analyze the differences in abundance of these cell types between imatinib-resistant and imatinib-sensitive TMEs. For example, Treg cells and proliferating Tregs are found to be more abundant in the imatinib-resistant TME. These immunosuppressive cells express high levels of TIGIT, an immune checkpoint molecule, establishing cellular communication with various tumor cells (such as DUSP1_fib, BASP1_fib, PDGFRA_fib, and IDO1_fib) expressing NECTIN2, thereby facilitating immune evasion of the tumor cells. Significantly, communication through the TIGIT-NECTIN2 pathway is more pronounced in the imatinib-resistant TME compared to the imatinib-sensitive TME. This observation suggests that the TIGIT-NECTIN2 interaction may contribute to imatinib resistance in GIST. Furthermore, myeloid cells in the TME play an immunosuppressive role. In the imatinib-resistant TME, there is an increase in the quantity of IDO+ DCs, which exhibit elevated expression of BTLA, another immune checkpoint molecule. These IDO+ DCs engage in interactions with various myeloid cells expressing TNFRSF14. However, it is important to note that the intensity of these interactions does not reach a significant level in the imatinib-sensitive TME (see Fig. 6f). We hypothesize that the emergence of imatinib resistance in GIST may trigger immune escape mechanisms within the tumor microenvironment (TME). Consequently, prospective immunotherapeutic approaches designed to target immune escape, such as interventions involving the TIGIT-NECTIN2 and BTLA-TNFRSF14 pathways, may offer novel and promising clinical treatment options. This substantiates our belief that the activation of immune checkpoint pathways contributes to imatinib resistance, suggesting that new immune checkpoint inhibitors could offer renewed hope for patients resistant to imatinib.

However, there are some limitations deserving attention. First, the sample size is relatively small because it is difficult to collect surgical samples of drug-resistant tumors due to the low incidence of GIST. In particular, only one patient contained both primary and metastatic tumors, which limited the analysis of tumor heterogeneity and evolution. Second, the tumor microenvironment can be influenced by a variety of factors, and the use of some other drugs might introduce certain biases in the detection results. In our study, 2 patients underwent third-line treatment. Considering the rapid progression of the tumor after imatinib resistance, these 2 patients had a very short duration of second and third-line treatments, and we believe this will not have a major impact on the main conclusions of the paper. Third, although we revealed the function and intercellular communication of some cell types based on the gene expression data, the results should be further evidenced by functional verification. Initially, we also tried to use bulk RNA from public databases for preliminary verification, but the mRNA data of sensitive tumors compared to resistant tumors is extremely scarce, limiting our further analysis. The related targets await further verification using cytology, functional studies, and animal experiments. In any case, all the samples were tested by multipoint sampling to better demonstrate the cell composition of tissues and the heterogeneity of gene expression profiles.

To sum up, based on the scRNA-seq analysis, we expounded on the cell ecosystem’s heterogeneity and dynamic properties in advanced GIST, revealed the immunosuppressive microenvironment in imatinib-resistant patients, and explored the complex interaction between drug-resistant tumors and their microenvironments. The findings of this study offer valuable resources to enhance our understanding of the mechanisms underlying imatinib resistance. This information can aid in developing more effective immunotherapy targets capable of reversing this resistance.

## Methods

### Sample collection

The Department of General Surgery at the Affiliated Hospital of Qingdao University provided nine tissue samples from seven patients diagnosed with GISTs. All nine samples were confirmed to be GISTs based on the National Comprehensive Cancer Network (NCCN) clinical practice guidelines.

Compared to other malignant tumors, GIST is a relatively indolent tumor, and many patients, even with widespread metastasis, can still have a longer survival with medication. Most metastasized tumors occur several years after the high-risk patients have had radical surgery to remove the primary tumor. However, patients who are initially diagnosed with primary tumors accompanied by metastasis are extremely rare. For these patients, the preferred treatment is targeted therapy mainly with imatinib. Even after resistance to multiple lines of therapy, it is very rare for patients to ultimately receive surgical treatment, which is due to the difficulty of surgery and poor treatment outcomes. The NCCN guidelines also recommend feasible surgical treatment only for patients with localized resistance. Therefore, it is difficult to simultaneously collect primary and metastatic tumors. Our research focuses on the tumor immune microenvironment of late-stage GIST patients, especially those who are resistant to treatment. Furthermore, we recognize that incorporating an analysis of the surrounding normal tissue alongside the tumor could enhance the paper’s content and provide stronger support for its conclusions. However, due to the high cost of scRNA-seq analysis, we decided not to collect normal tissue and instead increased the number of cases of resistant and sensitive tumors, hoping to enhance the persuasiveness of the inter-group comparison and more convincingly confirm the conclusions of the paper. We explored the tumor immune microenvironment of late-stage GIST from aspects such as tumor evolution, transcriptional heterogeneity, immune microenvironment analysis, and cell communication, and performed a differential comparison between resistant and sensitive tumors.

Because obtaining samples from late-stage GISTs posed challenges, we included all tumors classified as locally advanced or advanced based on preoperative and intraoperative assessments. In total, we gathered 9 samples, with each sample being collected from a minimum of 6 distinct sites within the tumor. These samples were subsequently combined into a single preservation solution tube and promptly dispatched to the laboratory for processing. Detailed clinicopathological characteristics of the patients are provided in Supplementary Table 1. Samples M-P01 and M-L01 are from the same patient’s peritoneal metastasis and liver metastasis, respectively. P-C07 and M-L07 are from the same patient’s small intestine primary tumor and liver metastasis, respectively. M-P01 and M-L01 are from a patient with small intestine GIST who relapsed 5 years after radical surgery, with liver and peritoneal metastases, and showed resistance to all three lines of targeted therapy. M-P02 is from a patient with small intestine GIST who relapsed 5 years after surgery, with peritoneal metastasis, and responded well to imatinib, with the tumor in a stable condition. M-P03 is from a patient with small intestine GIST who relapsed 1 year after radical surgery, with peritoneal metastasis, and showed resistance to all three lines of targeted therapy. P-G04 is a patient with primary gastric GIST, with a large tumor at a locally advanced stage, responding well to imatinib, and the tumor is in a stable condition. P-G05 is a patient with primary gastric GIST, with a large tumor at a locally advanced stage, responding well to imatinib, and the tumor is in a stable condition. P-G06 is a patient with primary gastric GIST, with a large tumor at a locally advanced stage, with a PDGFRA Exon 18 (D842V) mutation. Due to financial reasons, no targeted therapy was performed, and rapid tumor growth and rupture bleeding occurred during conservative observation. P-C07 and M-L07 are from a patient with small intestine primary tumor with liver metastasis. Imatinib treatment was effective, and the tumor is in a stable state.

The efficacy of imatinib treatment must be determined through radiological assessment, employing common diagnostic techniques such as abdominal enhanced CT, MRI, and PET-CT. According to the Choi or RECIST criteria, treatment sensitivity is typically indicated by complete remission, partial remission, or disease stability, while disease progression signifies resistance to treatment. Moreover, evaluating treatment effectiveness necessitates a thorough clinical assessment, demanding skilled radiologists to make a comprehensive judgment based on factors like tumor volume and density. For instance, effective targeted therapy may result in internal necrosis and cystic changes within the tumor, potentially leading to an increase in tumor lesion volume rather than a decrease.

### Tissue dissociation and preparation for scRNA-seq

Fresh tumor lesions were stored in cold GEXSCOPETM tissue preservation solution (Singleron Bio Com, Nanjing, China) and transferred for processing on ice within 30 min after resection. The tissue samples were trimmed, washed with Hanks balanced Salt Solution (HBSS) three times and cut into 1–2 mm pieces. Samples were digested using 2 mL of GEXscopetM tissue dissociation solution (Singleron). After digestion, the cell suspension was filtered using a 40-µm sterile strainers and centrifuged at 1000 rpm for 5 min. Dissociated cells were pelleted and resuspended in 1 mL phosphate-buffered saline (PBS; HyClone, United States). Subsequently, red blood Cells were removed with 2 mL of GEXSCOPETM red blood cell lysis buffer (Singleron). The solution was then centrifuged at 500 rpm for 5 min and resuspended in PBS. The samples were dyed with trypan blue solution (Sigma, United States) and observed under the phase contrast light microscope.

### Library preparation and scRNA-seq

The scRNA-seq libraries were constructed using the GEXSCOPER Single-Cell RNA Library Kit (Singleron Biotechnologies), according to the manufacturer’s protocol [57]. Single-cell suspensions with 1 × 10^5^ cells/mL in concentration in PBS (HyClone) were prepared. Single-cell suspensions were loaded in microfluidic devices. Subsequently, individual libraries were diluted to a final concentration of 4 nM. Then all these libraries were pooled and sequenced on Illumina HiSeq X with 150-bp paired-end reads.

### scRNA-seq quantifications

The sequencing data was processed using a standard internal pipeline to generate gene expression profiles. Initially, read one without poly T tails was filtered, and cell barcode and UMI were extracted. After trimming adapters and poly-A tails with fastp V1, read two was aligned to GRCh38 with ensemble version 92 gene annotation using fastp 2.5.3a and featureCounts 1.6.2 [58]. Next, reads with the same cell barcode, UMI, and gene were grouped to calculate the number of UMIs per gene per cell. The UMI count tables of each cellular barcode were used for further analysis. Cell type identification and clustering analysis were conducted using the Seurat program (v.3.0.1), an R package for scRNA-seq analysis [59]. The UMI count tables of each cellular barcode were loaded into R with the read.table function. For further clustering analyses, we set the parameter resolution to 0.6 for the FindClusters function to screen highly variable genes. To annotate the cell clusters, we identified differentially expressed genes (DEGs) between different groups or consecutive clusters using the FindMarkers function in Seurat. Finally, the identified gene clusters were annotated based on the expression of canonical marker genes. To perform GO functional enrichment analysis of gene sets and identify biological functions or pathways significantly related to specific expressed genes, we used the clusterProfiler software [60].

### Single-cell copy-number variation (CNV) and clonality analysis

Initial CNVs for each cell in the fibroblast and smooth muscle cells were estimated with the inferCNV package of R (version1.10.1; https://github.com/broadinstitute/inferCNV), using T cells as the reference. Firstly the cells were filtered with <2000 UMIs, and the inferCNV analysis was performed with parameters including “denoise”, default hidden markov model (HMM) settings, and a value of 0.1 for “cutoff”. The default Bayesian latent mixture model was selected to identify the posterior probabilities of the CNV alterations in each cell with the default value of 0.5 as the threshold, to reduce the false positive. The “subcluster” method was performed to infer the subcluster cells based on the CNV values generated by HMM. Each p- or q-arm level change, either a gain or a loss, was simply converted to equivalent CNV based on its location, referring to the genomic cytoband information. After data conversion, subclones with identical arm level CNVs were collapsed and trees were restructured to represent subclonal CNV architecture. For data visualization, the UPhyloplot2 algorithm (https://github.com/harbourlab/uphyloplot2/issues /4) was conducted to automate the generation of intra-tumor evolutionary trees. The arm level CNV calls curated from the inferCNV HMM subcluster CNV predictions algorithm and the percentage of cells in each of the subclones were used as inputs. the arm length is proportional to the percentage of cells plus a spacer (circle diameter + 5 pixels).

### Trajectory analysis of single cells

The cell lineage trajectory of CD4+ T was inferred by using Monocle2 with DDR-Tree reduction method. Based on the expression matrix and metadata information stored in the Seurat object, a Monocle object was firstly created, and by using “differentialGeneTest” function, DEGs from each cluster was derived, and genes with a q-value < 1e−5 were set as the ordering genes for further analysis. Batch effect were eliminated during dimensionality reduction. After the cell trajectories were constructed, differentially expressed genes and signatures along the pseudotime were detected using the “differentialGeneTest” function.

### Cell-cell interaction analysis

To analyze cell-cell interactions between different cell types, CellChat was used to identify significant ligand-receptor pairs within imatinb-resistant and sensitive samples. The interaction score refers to the total mean of the individual ligand-receptor pair average expression values in the corresponding interacting pairs of cell types.

### Immunohistochemistry and multiplex immunofluorescence staining

Tissue sectioning and immunohistochemistry were performed on formalin-fixed and paraffin-embedded (FFPE) GIST specimens. 3-μm-thick sections were deparaffinized, rehydrated, and washed. Endogenous peroxidase activity was blocked with 3% hydrogen peroxide for 10 min. After microwave antigen retrieval, slides were incubated with 3% H_2_O_2_ solution for 10 min. Then, the sections were incubated with primary antibodies, horseradish peroxidase (HRP)-linked secondary Antibodies, and diaminobenzidine, respectively. Counterstaining was done with hematoxylin solution after reactions. Finally, two experienced pathologists independently evaluated staining results for Foxp3 (Servicebio, GB11093), BTLA (Boster, A03149), TNFRSF14 (Affinity, AB2838686) and NECTIN2 (Boster, A08081-2).

Multiplex immunofluorescence staining of FFPE specimens was performed using a multiplex immunohistochemical kit (Servicebio, G1215) according to the manufacturer’s instruction. Primary antibodies including Foxp3 (Servicebio, GB11093), TIGIT (Sigma-Aldrich, ZRB1454), IDO1 (Boster, PB9603), panCK (Abcam, AB7753) and Lysozyme (Abcam, Ab108508) antibodies, were sequentially applied. Then, the samples were incubated with horseradish peroxidase-conjugated secondary antibody at room temperature for 2 h. Nuclei were stained with 4’,6-diamidino-2-phenylindole (DAPI; Servicebio, G1012). Multispectral images were obtained using the Olympus fluorescence microscope (Nikon Eclipse C1, Japan) and the confocal microscope (Nikon Eclipse E100, Japan).

### Statistical analysis

The statistical analysis was performed using R software (4.0.3) and GraphPad Prism software 7. All continuous data were shown as standard deviation (SD). An unpaired Student’s *t* test and one-way analysis of variance (ANOVA) were used for comparisons in two or more groups, respectively. Two-tailed *P* values of 0.05 or less were considered statistically significant.

### Supplementary information

All supplementary Figures and supplementary data 1

Supplementary figure and table legends

Supplementary Data 2

Supplementary Data 3

Supplementary Data 4

checklist_AJ

## Data Availability

All sequencing data generated for our manuscript have been deposited in publicly accessible databases. The single-cell RNA sequencing data generated in this study are available in the National Center for Biotechnology Information (NCBI) Gene Expression Omnibus (GEO) database under the accession code GSE254762. All relevant data supporting the key findings of this study are available within the article and its Supplementary Information files or from the corresponding author upon reasonable request.
