# Supplementary material for: Deciphering the tumor immune microenvironment of imatinib-resistance in advanced gastrointestinal stromal tumors at single-cell resolution
Source: Cell Death Dis. 2024 Mar 5;15(3):190. doi: 10.1038/s41419-024-06571-3 (PMC10914684; doi:10.1038/s41419-024-06571-3)
Supplement: Supplementary file 1 — All supplementary Figures and supplementary data 1 [file 41419_2024_6571_MOESM1_ESM.pdf]

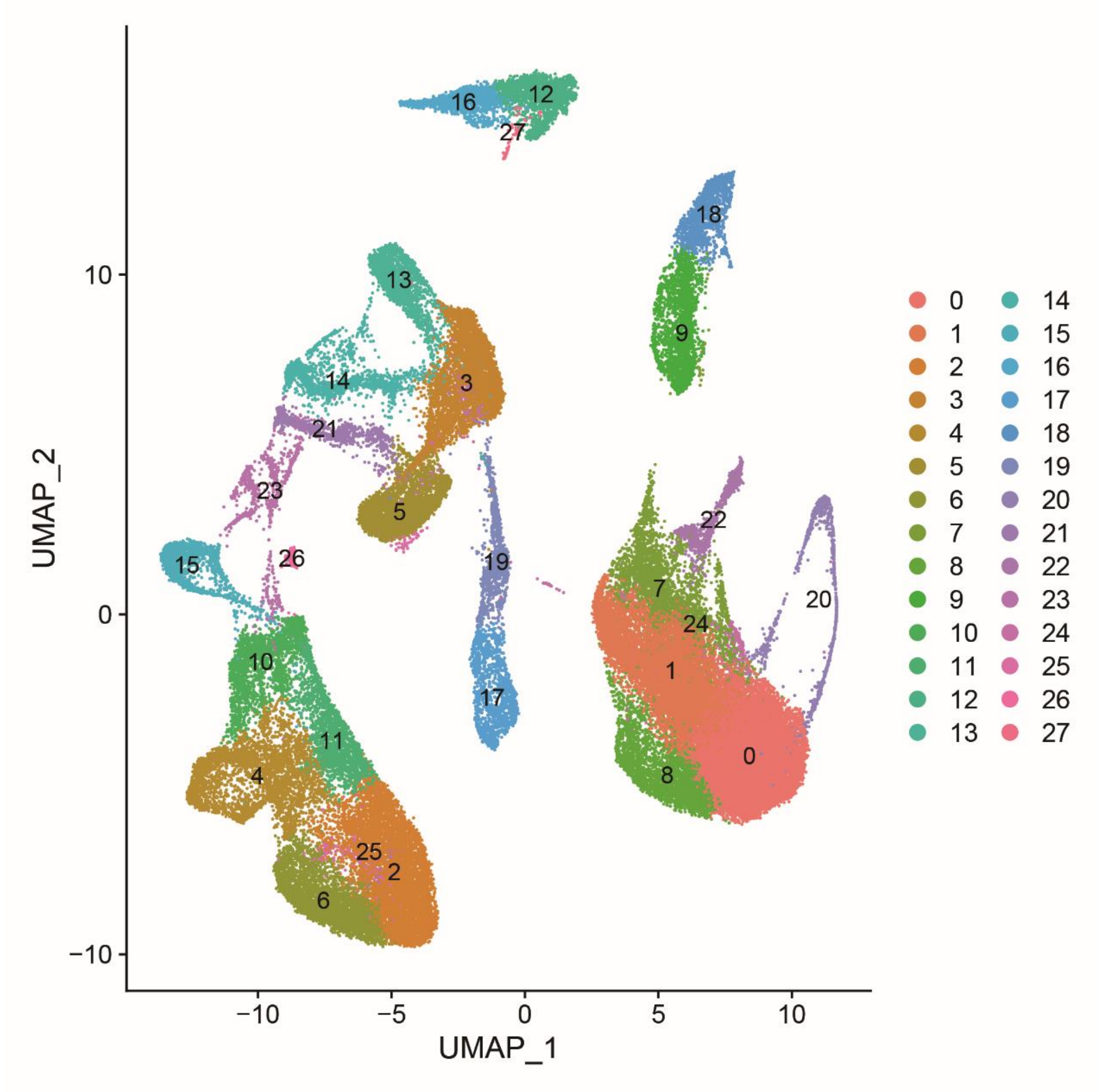

Supplementary Figure 1. UMAP plot of all cells showing different clusters.

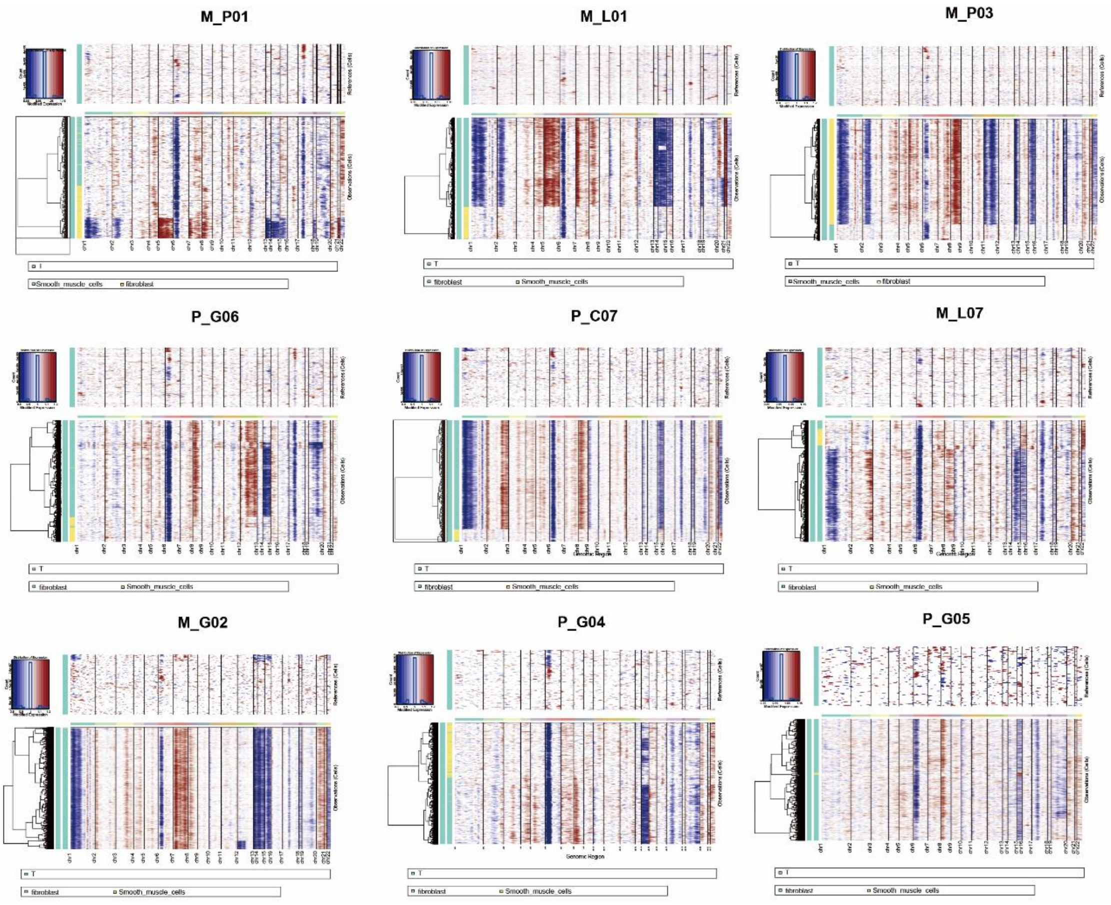

Supplementary Figure 2. The hierarchical heatmap showing large-scale CNVs in fibroblast cells and smooth muscle cells form each sample. The branches are delineated according to the percentage of cells in the subclone containing the corresponding CNVs. The canonical CNV events in each lesion were labeled in the clonality tree. The subclones in red background indicated the shared subclone between two samples from the same pati

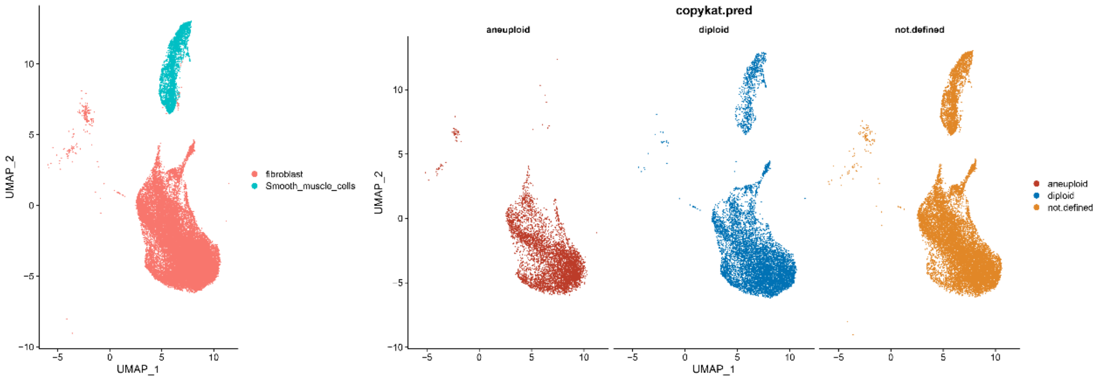

Supplementary Figure 3. Identification of the malignance of smooth muscle cells by copyKAT algorithm.

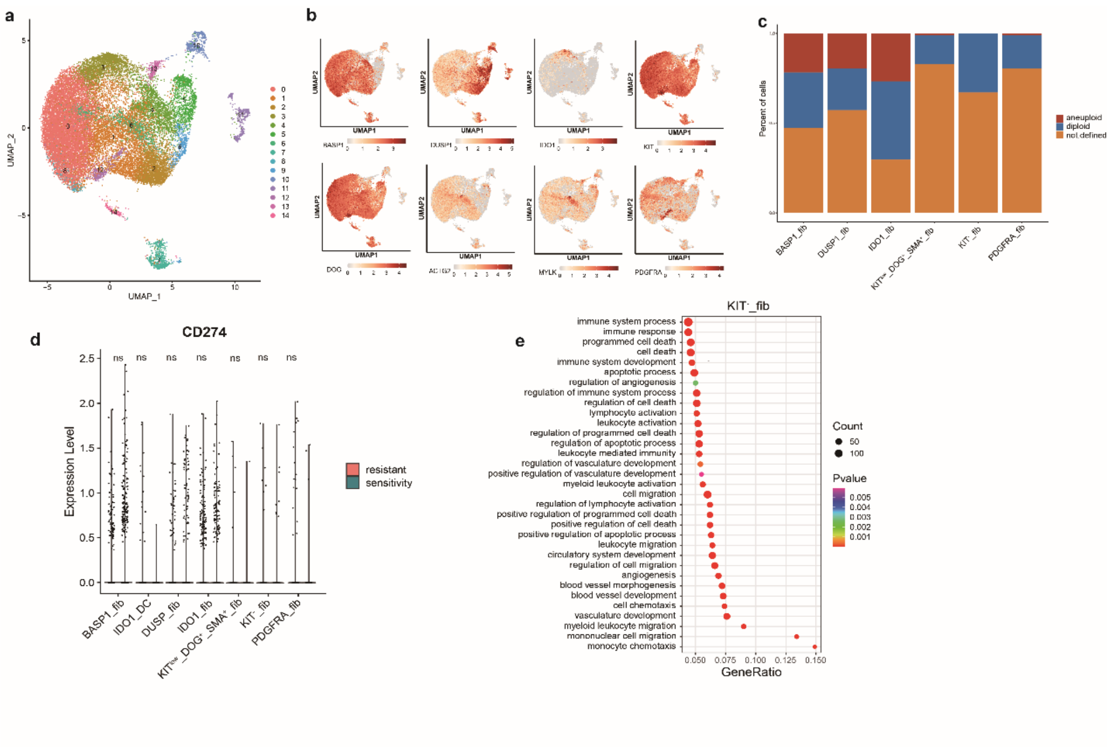

Supplementary Figure 4. Distinct clusters of malignant cells in GIST.

(a) 15 subclusters of fibroblast cells were identified by UMAP analysis.

(b) Marker gene expression of each cell type.

(c) Expression of CD274 (PD-L1) in each cell type.

(d) The ploidy analysis of fibroblast cells by copyKAT algorithm.

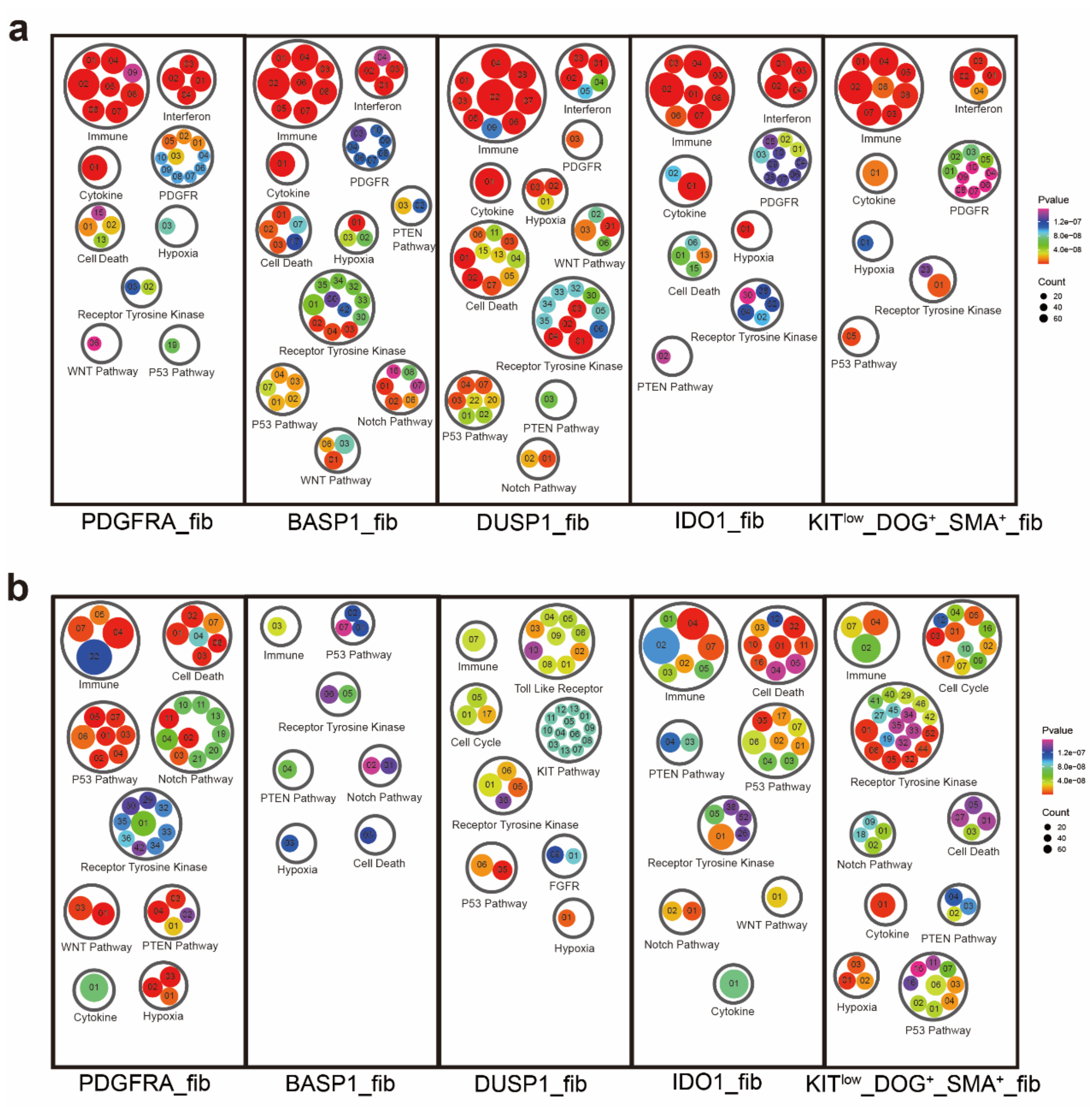

Supplementary Figure 5. Reactome enrichment analysis of DEGs in each malignant cell type between imatinib resistant and sensitive patients. a Reactome enrichment analysis of up-regulated DEGs between imatinib resistant samples and imatinib sensitive samples. **b** Reactome enrichment analysis of down-regulated DEGs. The color in each dot showed the p-value of each pathway. The size for each dot showed the enriched gene number in each pathway. The number in each dot showed the number for each pathway corresponding to Supplementary Data 3.

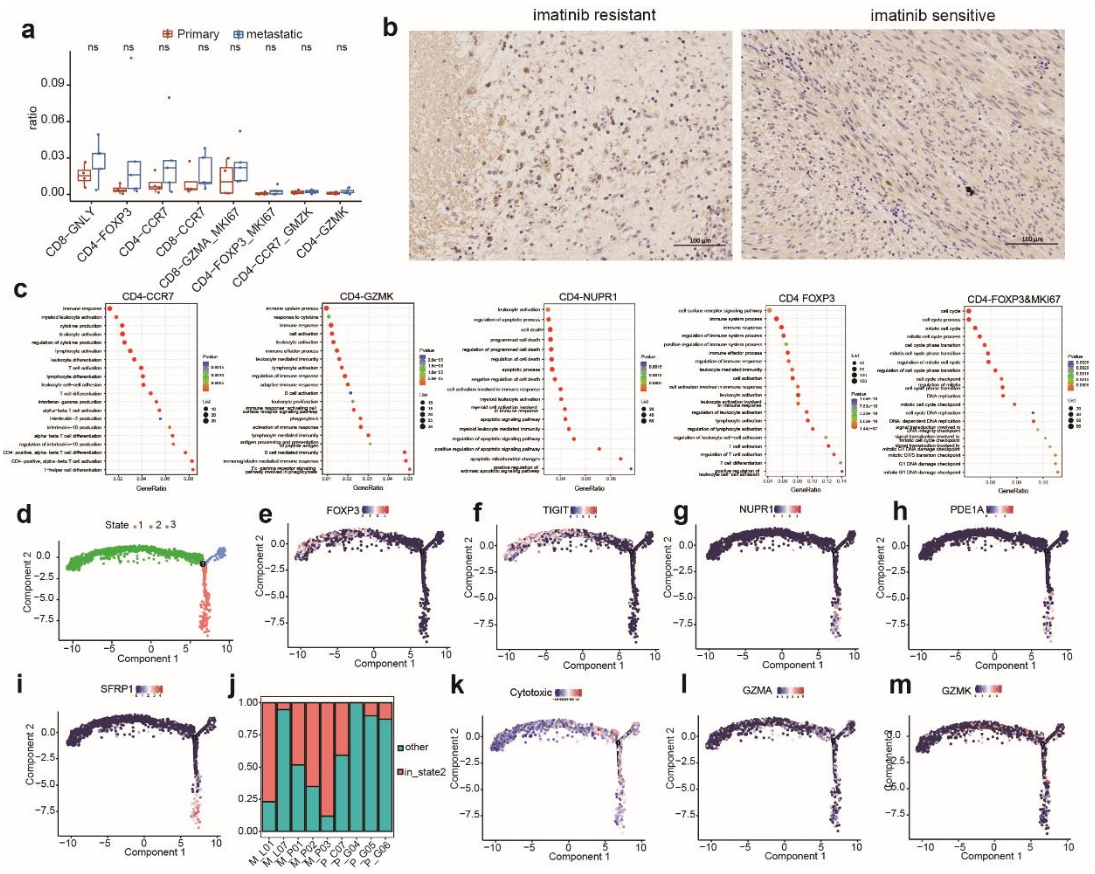

Supplementary Figure 6. The transcriptional heterogeneity of T cells.

(a) Comparison of cell count proportion of each cell type between primary and metastatic patients.

(b) IHC analysis of FOXP3 on GIST samples.

(c) GO enrichment analysis of each CD4 cell type.

(d) The trajectory plot colored by different state.

(e-i) The expression level along trajectory plot of FOXP3 (e), TIGIT (f), NUPR1 (g), PED1A (h) and SFRP1 (i).

(j) Cell count proportion of CD4-CCR7 cells in state 2 and other states.

(k) The cytotoxic signature expression along trajectory pathway.

(l, m) The expression level along trajectory plot of GZMA (l) and GZMK (m).

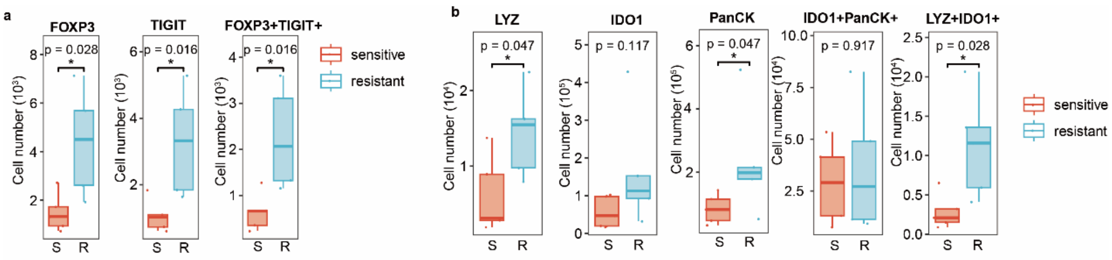

Supplementary Figure 7. Comparison of positive cells and co-localized positive cells count between imatinib resistant and sensitive patients. The Halo v3.0.311.314 analysis software was used to quantify the number of positive cells and co-localized positive cells in each section.

(a) The expression of FOXP3 (red) and TIGIT (green) in imatinib resistant and sensitive patients.

(b) The expression of panCK (red), LYZ (green) and IDO1 (magenta) in imatinib resistant and sensitive patients.

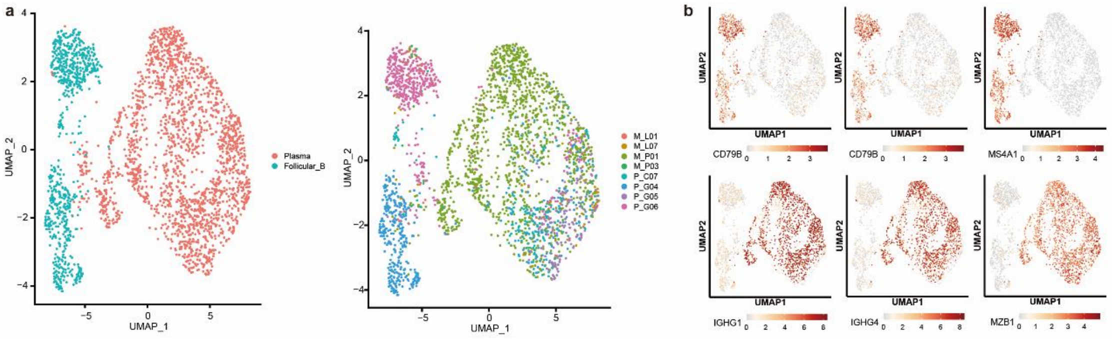

Supplementary Figure 8. The transcriptional heterogeneity of B cells.

(a) 2 subclusters of B cells were identified by UMAP analysis.

(b) Marker gene expression of each cell type.

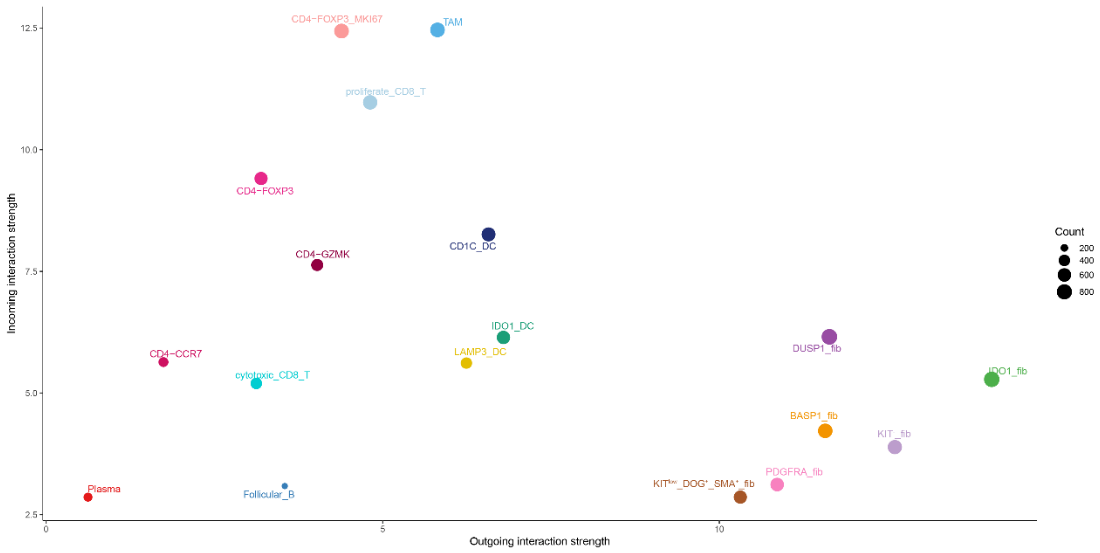

Supplementary Figure 9. The overall cell communication strength between each cell type.

Supplementary Data 1 Clinicopathologic information of 9 advanced gastrointestinal stromal tumor samples

	M-P01	M-L01	M-P02	M-P03	P-G04	P-G05	P-G06	P-C07	M-L07
Sex	Male	Male	Male	Male	Male	Female	Female	Male	Male
Age (years)	55	55	62	69	58	63	67	66	66
Tumor stage	Advanced	Advanced	Advanced	Advanced	Locally advanced	Locally advanced	Locally advanced	Advanced	Advanced
Tumor site	Peritoneal metastasis	Liver metastasis	Peritoneal metastasis	Peritoneal metastasis	Stomach	Stomach	Stomach	Small intestine	Liver metastasis
Recurrence	Yes	Yes	Yes	Yes	No	No	No	No	No
Primary tumor site	Small intestine	Small intestine	Small intestine	Small intestine	/	/	/	/	/
Mitotic index (/50 HPF)	>5	>5	<5	>50	<5	<5	<5	<5	<5
Lines of targeted treatment	Progression after 3 lines	Progression after 3 lines	First-line	Progression after 3 lines	First-line	First-line	No	First-line	First-line
Imatinib resistance	Yes	Yes	No	Yes	No	No	Yes (Primary resistance)	No	No
Gene mutations	KIT exon 9	KIT exon 9	KIT exon 11 and exon 17	KIT exon 11 and exon 13	KIT exon 11	KIT exon 11	PDGFRA exon 18 (D842V)	KIT exon 11 and exon 18	KIT exon 11 and exon 18
